# More than just the mental health act – foundation-specific teaching to inspire psychiatrists

**DOI:** 10.1192/bjo.2021.397

**Published:** 2021-06-18

**Authors:** Robert Heminway, Lauren Fitzmaurice, Hamid Alhaj, Edward Fearnley

**Affiliations:** Sheffield Health and Social Care NHS Foundation Trust

## Abstract

**Aims:**

This project aimed to further develop a teaching programme for Foundation Doctors attached to a psychiatry rotation. The purpose was threefold – to educate foundation doctors about important psychiatric topics; to encourage them to think about wider impacts of psychiatry; and to inspire them to consider psychiatric training in the longer term.

**Background:**

The Royal College of Psychiatrists’ mission statement includes actively promoting psychiatry as a career and improving knowledge of mental health, including its interactions with people's physical and social backgrounds. Targeting foundation doctors rotating into psychiatry posts is a good opportunity to achieve these objectives, as they will be the cross-speciality doctors of the future, and have specific learning needs given their unique rotations and new medical careers.

**Method:**

On one Wednesday morning per month Foundation Doctors had a specific teaching session for them. The sessions consisted of four 30-minute teaching blocks which, crucially, were given by foundation doctors. They were facilitated by a core psychiatry trainee, and the topics were decided by the doctor teaching each 30-minute block. The foundation doctors were able teach on any topic related to psychiatry that interested them. Feedback forms were developed and provided at the end of each session for the foundation doctors, as well as at the end of each recent foundation rotation, to get feedback on the overall quality of the course delivered.

**Result:**

The programme has now had 6 complete cohorts of foundation doctors. We have built a varied topic bank from past sessions, including the Mental Health Act, dementia, the Mind-Body Problem, psychiatry in video games and sociology of psychiatric illness, amongst other topics. All foundation doctors questioned have agreed or strongly agreed that the sessions were helpful for their psychiatric rotation and general medical training. Particularly praised aspects were the ability to discuss psychiatric topics that weren't normally discussed in an academic environment, being able to take ownership over learning and practicing giving teaching. Vitally, core trainee facilitators also found the sessions inspiring for their training.

**Conclusion:**

The Foundation Teaching Programme has increased doctors’ knowledge of a range of psychiatric topics, the breadth of which and agency in choosing topics has increased engagement with psychiatry, regardless of planned medical training speciality. Areas to explore in the future include potentially opening attendance to medical students and physician associate students, and to other regions of the deanery. Evaluating the long-term impact of this training is also warranted.

